# Adherence to medication, physical activity and diet among older people living with diabetes mellitus: Correlation between cognitive function and health literacy

**DOI:** 10.1016/j.ibror.2020.07.003

**Published:** 2020-07-08

**Authors:** Thaísa Soares Crespo, João Marcus Oliveira Andrade, Deborah de Farias Lelis, Alice Crespo Ferreira, João Gabriel Silva Souza, Andréa Maria Eleutério de Barros Lima Martins, Sérgio Henrique Sousa Santos

**Affiliations:** a Laboratory of Health Sciences, Montes Claros State University, Montes Claros, Minas Gerais, Brazil; b Department of Physiopathology, Center of Health and Biological Sciences, Montes Claros State University, Montes Claros, Minas Gerais, Brazil; c Department of Nursing, Center of Health and Biological Sciences, Montes Claros State University, Montes Claros, Minas Gerais, Brazil; d Piracicaba Dental School, University of Campinas, Piracicaba, Brazil; e Department of Dentistry, Center of Health and Biological Sciences, Montes Claros State University, Montes Claros, Minas Gerais, Brazil; f Institute of Agricultural Sciences. Food Engineering College, Federal University of Minas Gerais (UFMG), Montes Claros, Minas Gerais, Brazil

**Keywords:** Cognition, Mini-mental state examination, Literacy, Elderly, Therapeutic adherence

## Abstract

**Background:**

Diabetes mellitus (DM) is a public health problem, which requires enhanced self-care in order to avoid complications. However, cognitive impairment can reduce these abilities and may affect health literacy (HL) of patients in terms to understand and apply information. Therefore, this study evaluated the correlation between cognitive condition and HL related to medication adherence, physical activity and nutritional status among people living with DM.

**Methods:**

A cross-sectional study was carried out among elderly people (≥ 60 years old) with DM. The cognitive condition was evaluated using the Mini-Mental State Examination (MMSE) and the HL using the following questionnaires: Literacy Assessment for Diabetes (LAD-60), Nutritional Literacy among People with Diabetes (NLD), Health Literacy on the Practice of Physical Activities among Diabetics (HLPPA - D), and Health Literacy regarding Drug Adherence among Diabetics (HLDA-D). Sociodemographic and biochemical profile was also evaluated. Spearman correlation was used (p < 0.05).

**Results:**

187 individuals with DM were included. Regarding laboratory analyses, insulin dosage had a mean value of 12.3 microUI/mL (SD: ±15.7), mean blood glucose was 148.1 mg/dl (SD: ±59.7) and mean HbA1c was 7.54 % (SD: ±1.8). In the correlation analysis, higher age and lower income were weakly correlated with lower cognitive level. No correlation was identified for biochemical variables and cognitive condition. A positive and weak correlation between cognition and HL was observed in the studied population.

**Conclusions:**

In older people living with DM the cognitive condition is correlated to specific topics of HL (nutritional status, physical activity and medication adherence).

## 1 Introduction

Diabetes Mellitus (DM) is an important public health concern worldwide, which has been associated with environmental and genetic factors (Mambiya et al., 2019). In 2019, the International Diabetes Federation estimated that 463.0 million adults (20–79 years) were living with DM in the world (Mambiya et al., 2019). Although the burden of disease is already high, the projection for 2030 is that 578.4 million adults and, in 2045 that 700.2 million adults in the same age group will be affected by DM. However, this projection may be much higher considering the significant increase in the risk factors (Mambiya et al., 2019).

DM is characterized by a metabolic disorder resulting from the breakdown of glycemic homeostasis with persistent hyperglycemia. This occurs due to insulin production deficiency, and/or reduced insulin sensitivity associated to impaired insulin receptor signaling, which produce acute and chronic complications (Stumvoll et al., 2005; American Diabetes Association, 2010). Interestingly, type 2 diabetes, which represents ≈90 % of cases, is preventable since its main risk factors are related to body conditions, such as physical inactivity, diet and overweight/obesity (Jorge et al., 2017; Chatterjee et al., 2018; International Diabetes Federation, 2019; Batista-Jorge et al., 2020). Even among diagnosed patients, these conditions need to be controlled as part of treatment and to avoid complications related to diabetes (International Diabetes Federation, 2019). Therefore, an essential part of the DM management should include health literacy (HL) of patients, since this concept consider the importance of the patient to understand and apply information regarding the disease and its complications (Bailey et al., 2014). These abilities could improve patients’ behaviors to reduce the risk of complications, such as medication adherence and healthy lifestyle, and improve DM treatment (Bailey et al., 2014).

Previous studies have shown the association between DM literacy and healthy behaviors, such as medication adherence, better nutritional status, regular use of insulin or other drugs, and physical activity (Lee et al., 2016; Yeh et al., 2018). Importantly, these results suggest an improvement in the patient’s health self-care, which is essential to reduce morbi-mortality burdens in the DM treatment (Lee et al., 2016; Yeh et al., 2018). In contrast, scientific evidence suggests that people living with DM and poor HL have a higher risk to develop cognitive impairments, such as Alzheimer's disease and cognitive disorders (Bruce et al., 2003). As well DM, cognitive impairments have a high prevalence in the world and may affect 42 million people in 2020 and 81 million in 2040, and this increasing has been associated with the prevalence of DM (Ferri et al., 2005).

In the evaluation of cognitive impairments, the Mini-Mental State Examination (MMSE) is the most used tool for the initial assessment of cognition level. This questionnaire evaluates the orientation, memory, attention, language and visual-spatial skills related to cognitive condition (Folstein et al., 1975; Almeida, 1998; Scazufca et al., 2009). Therefore, it is expected that people living with DM have a properly cognitive condition to achieve a better level of HL and, consequently, improve self-care. In fact, previous study identified the association between HL and cognitive condition among older adults living with DM (Nguyen et al., 2013). However, since the management of diabetes should include healthy behaviors related to medication adherence, physical activity and nutritional status, the association between cognitive condition and HL related to these aspects among people living with DM has not been widely evaluated. Moreover, the early screening of cognitive condition and HL levels could enhance the DM management by health professionals (Mendes et al., 2019). Thus, this study evaluated the correlation between cognitive condition and socio-demographic, clinical conditions, and HL among people living with DM. It is expected that better cognitive condition be positively correlated with high HL levels.

## 2 Material and methods

### 2.1 Ethical considerations

This study is part of a research project - Impact of educational actions on HL levels among elderly people registered in the Family Health Strategy: a randomized trial - which presents as one of the goals to create HL instruments for people with DM. It was carried out in accordance with the ethical principles of the Declaration of Helsinki and approved by the Ethics Committee of the State University of Montes Claros. CAAE: 34687414.0.0000.5146. Opinion Number: 764,743. Reporting Date: 9/19/2014.

### 2.2 Sample

A cross-sectional study was carried out (2017–2018) among elderly people with a diagnosis of DM attended by the public health service of a medium-sized city (estimated population: 400,000 inhabitants) in the southeastern region of Brazil (Paim et al., 2011). All individuals that attended inclusion and exclusion criteria, accepted to participate in the study and were present in two services randomly selected from public health system (primary care) in the city, were included.

The following inclusion criteria were considered: 1) registered in the service; 2) ≥ 60 years old; 3) diagnosed with DM according to the American Diabetes Association (fasting plasma glucose ≥ 126 mg / dL (7.0 mmol / L), 2 h of plasma glucose ≥ 200 mg / dL (11.1 mmol / L) during an oral glucose tolerance test performed according to the description of the World Health Organization, glycated hemoglobin (HbA1c) ≥ 6.5 % (48 mmol / mol), or in patients with classic symptoms of hyperglycemia or hyperglycemic crisis, a random plasma glucose ≥ 200 mg / dL (11.1 mmol / L) (American Diabetes Association, 2010, 2018).

The exclusion criteria were: 1) Not having Portuguese as native language; 2) special needs that do not allow properly communication such as decreased or lost visual/auditory acuity (reported or perceived); 3) use of any drug or alcohol at the time of the interview; 4) neurological diseases with cognitive impairment and dementia of non-vascular etiologies (dementia by Lewy bodies, dementia associated with Parkinson's disease, frontotemporal dementia, Alzheimer's dementia and Creutzfeldt-Jakob dementia). Respecting these criteria, participants were evaluated until reaching the "n" of the sample calculation necessary for the study.

#### 2.2.1 Sample size

Since no previous study estimated the number of elderly people with DM in the city evaluated, the sample size was calculated by using a sampling procedure for infinite population that considered the following parameters: p = 0.5; q = 0.5; E = 0.075 and Z = 1.96, (q = 1 - p value; E = sampling error; Z = constant of 1.96) using the formula $n=\;\frac{Z^{2}\propto /2^{\;.\;p\;.\;q}}{E^{2}}$ (Triola, 1999). The non-response rate was considered to be 10 %, totaling a population sample of 186 elderly people with DM.

### 2.3 Procedures

After signing the informed consent, the individuals were evaluated and interviewed by a researcher at the health care service unit, called Family Health Strategy (FHS), on days and times previously scheduled or, through active search, at their own home when they did not come to the service. The following variables were considered: sociodemographic characteristics (age, gender, skin color or self-report race, marital status, education and per capita income), blood samples for biochemical tests (glycated hemoglobin, insulin and plasma glucose), the Mini-Mental State Examination (MMSE) and questionnaires to assess health literacy (HL) with a focus on literacy in medication adherence, diet and physical activity. The assessment of individuals was standardized to reduce the effect on our results. All individuals were submitted to blood draw that was conducted in the morning after 8 h on fasting. Moreover, previous instructions were provided for all individuals to avoid physical activity before blood draw and the use of any medication that could affect biochemical analysis.

### 2.4 Measures

#### 2.4.1 Analysis of sociodemographic profile

The sociodemographic aspects evaluated were the age (≤ 63, 64−67, 68−72 and ≥ 73), the gender (male and female), skin color or self-report race (white and non-white), the marital status (single or with partner), the education (0, 1−4, 5−8 and ≥ 9 years) and the per capita income (> 291, 202−291, 134−201 and < 134 dollars) by the description of the absolute and relative frequencies for the categorical variable and of the mean, standard deviation and maximum and minimum values for continuous variables.

#### 2.4.2 Biochemical analysis

Serum measurements of glucose, glycated hemoglobin (HbA1c) and insulin were performed using a blood sample. All analyses were performed at the same laboratory. Glucose measurement was performed using the enzymatic colorimetric method, with values between 70 and 99 mg/dl being considered as reference. The measurement of glycated hemoglobin was performed by high-performance liquid chromatography (HPLC) and the results were dichotomized into good controls (HbA1c < 7%) and poor controls (≥ 7%). The insulin dosage was done by chemiluminescence and the reference value was 1.90–23.00 microUI/mL. Data were expressed as mean and standard deviation. The HOMA (Homeostatic Model Assessment for Insulin) index was also evaluated considering glucose x insulin / 22.5.

#### 2.4.3 Cognition assessment: mini-mental state examination (MMSE)

MMSE is a screening tool for cognitive assessment developed by Folstein in 1975. The tool evaluates five areas of cognitive function including orientation, immediate memory, attention and calculation, language and verbal construction (evocation memory) (Folstein et al., 1975). The total score ranges from 0 (impaired) to 30 (normal) and several cutoff points have been used for the application of MMSE depending on the number of years of education (Brucki et al., 2003). In the present study, in accordance with the suggestions for the use of the questionnaire in Brazil, it was adopted the cutoff point of "20″ for individuals with no education and "24″ for individuals with some education (Almeida, 1998; Brucki et al., 2003).

#### 2.4.4 Health literacy in diabetes

Some questionnaires were used to measure overall HL and others focusing on the HL related to nutrition, physical activity and medication adherence, using previous developed questionnaires (Souza et al., 2006; Eleutério et al., 2018; Martins et al., 2018; Cardoso et al., 2019).

##### 2.4.4.1 Literacy assessment for diabetes (LAD-60)

LAD-60 was developed and validated in the English language and consists of 60 words to be read by people with DM in order to assess HL (Nath et al., 2001). The questionnaire showed a properly cross-cultural adaptation for the Brazilian Portuguese version and was used as a screening tool to identify HL levels among people with DM. The 60 words are distributed in three lists of 20 words that must be read by the researcher in front of the interviewer that evaluates if the pronunciation is accurate and, therefore, the score can vary from 0 to 60 (Nath et al., 2001; Neto et al., 2018). The cutoff point adopted was the low-limit of the confidence interval.

##### 2.4.4.2 Nutritional literacy among people with diabetes (NLD)

The evaluation of HL related to nutritional habits was assessed by the Nutritional Literacy among People with Diabetes (NLD) questionnaire whose interpretability is made by identifying and counting the correctness of the associations between 24 trios of words (correct/incorrect/I don't know), with scores from 0 to 24 (cut ≤ 18). Therefore, scores > 18 indicate adequate literacy (75 % of associations are correct) and scores between 0 and 18 indicate inadequate NLD (Eleutério et al., 2018).

##### 2.4.4.3 Health literacy on the practice of physical activities among diabetics (HLPPA-D)

For the evaluation of HL regarding the physical activity practice among people living with DM, a questionnaire was used based on the interpretability of the identification and counting of the correctness of the associations between 18 trios of words (correct/incorrect/I don't know), with scores from 0 to 18 (cut ≤ 14). Thus, scores > 14 indicate literacy regarding the practice of physical exercise and scores between 0 and 14 indicate illiteracy (Martins AMEBL, 2018).

##### 2.4.4.4 Health literacy regarding drug adherence among diabetics (HLDA-D)

To assess HL related to drug adherence among people living with DM, a questionnaire called HLDA was used to assess the skills of association and understanding of the most common medical terminology used for DM (Cardoso et al., 2019). It includes 18 words related to DM and its treatment, with scores from 0 to 18 (cut ≤ 14). Thus, scores > 14 indicate literacy regarding adherence to drug treatment and scores between 0 and 14 indicate illiteracy (Cardoso et al., 2019).

### 2.5 Statistical analysis

The sample was described as absolute and relative frequencies for the categorical variables, and mean/standard deviation for the quantitative variables. The normality of data was checked by Kolmogorov-Smirnov test. Correlation among socio-demographic/clinical conditions and cognitive impairment, and among health literacy and cognitive impairment was conducted by Spearman test. The significance level was set as p < 0.05. The effect size of correlation was interpreted according to Cohen, 1988 (Cohen, 1988), as: a weak/small effect of 0.2, a medium effect of 0.5 and a large effect of 0.8 (Lakens, 2013). Statistical analyzes were performed using the Statistical Package for the Social Sciences version 25 for Windows (SPSS).

## 3 Results

### 3.1 Sociodemographic and clinical characteristics of the sample

The final sample included 187 older people living with DM, with a mean age of 68.97 years (SD: ±7.17; minimum: 60, maximum: 92). Most of sample had 73 years or more (29.4 %), were women (68.4 %), non-white people (66.8 %) and with an average education time of 6.59 years (SD: ±4.75; minimum: 0, maximum: 28). Regarding the biochemical parameters, insulin dosage had a mean value of 12.3 microUI/mL (SD: ±15.7) and HbA1c was 7.54 % (SD: ±1.8) (Table 1). The average of HOMA index was 80.5 (SD: ± 122.4).

**Table 1:** Socio-demographic and clinical conditions of elderly people with DM from a medium-sized municipality in the southeastern region of Brazil.

Variables	mean (SD)
Age (years)	68.97 (±7.17)
Education (years)	6.59 (±4.75)
Insulin (microUI/mL)	12.3 (±15.7)
Glucose (mg/dL)	148.1 (±59.7)
HbA1c (%)	7.54 (±1.8)
	n (%)
Age	
≤ 63	51 (27.3)
64−67	44 (23.5)
68−72	37 (19.8)
≥ 73	55 (29.4)
Gender, n (%)	
Male	59 (31.6)
Female	128 (68.4)
Education	
0	22 (30.5)
1−4	61 (25.1)
5−8	47 (32.6)
≥ 9	57 (11.8)
Marital status, n (%) *	
Single	95 (51.1)
With partner	91 (48.9)
Race/Skin color	
White	62 (33.2)
Non-white	125 (66.8)
*Per capita* income (US$) **	
> 291	43 (23.0)
202−291	44 (23.5)
134−201	42 (22.5)
< 134	43 (23.0)
Cognitive impairment	
Present	35 (18.7)
Absent	152 (81.3)

* Variation in the number of individuals.

** R$ 1.00 was equivalent to US$ 3.23 at the time of this study.

### 3.2 Correlation between sociodemographic/clinical characteristics and cognitive condition

The variables age, education, and income showed significant correlation with cognition condition. Higher age, lower per capita income and the number of years of schooling, were correlated with lower MMSE scores. However, the effect size (r) shows a small/weak correlation among these variables. Regarding the serum levels of glucose, insulin and glycated hemoglobin, there was no significant correlation with the MMSE scores (Table 2). No correlation was identified between HOMA index and MMSE scores (p = 0.263).

**Table 2:** Correlation among sociodemographic/clinical conditions and cognitive impairment among elderly people living with DM.

Variables	Cognitive Function	
	r	p
Age	−.174	0.017*
Education	.477	0.000*
*Per capita* income	.186	0.014*
Insulin	−.105	0.197
Glucose	.059	0.451
HbA1c	−.032	0.679

* p < 0.05.

### 3.3 Correlation between health literacy on medication adherence, nutrition, physical activity, and cognitive condition

For the HL evaluation considering specific aspects, medication adherence, nutrition, physical activity, and cognitive condition, a positive and significant correlation was found among these questionnaires with higher MMSE scores. It suggests that better cognitive condition is correlated with higher HL in terms of medication adherence, nutrition, physical activity, and cognitive condition among elderly people living with DM (Table 3). However, the effect size shows a weak correlation among them.

**Table 3:** Correlation among health literacy and cognitive condition among elderly people living with DM.

Variables	Cognitive Function	
	r	p
LAD-60	.328	0.000*
NLD	.309	0.001*
HLDA - D	.358	0.000*
HLPPA - D	.297	0.000*

* p < 0.05.

## 4 Discussion

HL considers the development of personal, cognitive and social skills necessary to access, understand, evaluate and apply information related to health. For people living with DM, low HL level has been associated with low adherence to treatment and the development of complications (Sorensen et al., 2012). Previous studies suggest that several risk factors are associated with poor/non-adherence to DM treatment, including sociodemographic factors, clinical conditions, such as cognitive impairment, and factors related to the health system, which determine self-care practices among people with DM (Al-Hayek et al., 2012; Mendes et al., 2019). Interestingly, the results found here showed a correlation between better cognitive condition with higher level of HL considering important aspects of DM management, such as nutrition, physical activity and medication adherence. Although the correlations were weak, these results suggest the existence of relationship between cognitive condition and HL among people living with DM.

Cognitive impairment is common among elderly people with DM, but it is unclear to what extent cognitive function is associated with HL (Nguyen et al., 2013). Our hypothesis was that cognitive function is associated with HL and it was accepted. This suggests that poor cognitive function may reduce HL level. In fact, previous study evidenced that cognitive function indicators were significantly associated with HL, showing that and increase in unity in the MMSE score was associated with a 20 % increase in the chances of improve HL level (Nguyen et al., 2013). Thus, the relationship between cognition and HL deserves more attention from clinicians and public health, since both aspects can contribute to undesirable health outcomes (Federman et al., 2009).

The association between cognitive function and HL has not been widely evaluated, mainly in Brazil. Since people with low HL show a worse ability for verbal communication, mental processing and efficiency to understand text and numerical information, as well critical thinking, a relationship with low cognitive condition is expected (Federman et al., 2009). An improvement in the cognitive ability and, consequently, in the HL level may promote the self-care among people living with DM, especially elderly people which is naturally affected by cognitive impairment.

In the scientific literature, studies evaluating the correlation among HL, DM, cognitive condition and physical activity practice are scarce (Iwata and Munshi, 2009; Nguyen et al., 2013; Friis et al., 2016; Ueno et al., 2019). A previous study showed that HL had a direct positive effect on medication adherence and possibly an indirect and positive effect on physical activity practice and diet (Ueno et al., 2019). Furthermore, it has been shown that regular physical activity among adults and elderly optimizes cognitive condition (especially in those already with mild cognitive impairment) (Sanders et al., 2019). Interventions focusing to improve HL and physical activity practice have been able to enhance glycemic control among people living with DM (Liu et al., 2018). The practice of physical activity among people with DM is essential to achieve clinical goals and to prevent complications associated with the disease. However, some studies have shown that people with DM exercise less regularly compared to non-diabetics (Hamasaki, 2016; Joseph et al., 2016). A previous study evidenced that people who did not adhere to the practice of physical activity showed more cognitive impairment than the adherents; this impairment was attributed to less autonomy (Rothman et al., 2004). A previous meta-analysis described that HL interventions to improve physical activity among adults and the elderly with DM increase the frequency and duration of physical activity among people with high levels of HL enhancing glycated hemoglobin levels control (Lam and Leung, 2016). It is important to highlight that the aging process is directly related to changes in cognitive function, such as reduced memory and motor capacity, and older people are more likely to not understand health-related information (Gazmararian et al., 1999; Moura et al., 2019).

Education level is also an important factor that affects the HL level and cognitive function (Roundtable on Health Literacy et al., 2013). This fact can compromise the acquisition of knowledge, especially the level of health education (Estrada et al., 2004). Considering that elderly people can show lower education levels, compared to younger people, a poor HL level in this age group is expected. Therefore, this harmful combination of education level, HL and cognitive condition among elderly people suggests a profile of people more susceptible to morbidity and mortality related to the disease (Gazmararian et al., 1999; Apolinario et al., 2012), such as DM.

Another condition that may affect HL is income. The results are in accordance with the literature since families with lower income can have less access to the health services and information related to health and disease (Friis et al., 2016). Thus, in a low-income population living with DM, it is expected a lower glycemic control and, consequently, a higher probability of complications from the disease (Bains and Egede, 2011).

Despite the important results, the sample size included must be considered a limitation, since it was properly to identify the outcome and correlation analysis, but not for robust statistical analysis, such as logistic regression. Further studies should consider the correlation with cognitive condition according with the type of diabetes, as well to expand this area of research, including other potential risk factors for DM, such as specific diet (sugar intake) and alcohol use. Moreover, although HL questionnaires have properly psychometric properties and are easily and quickly applied, due the age of individuals some answers may be sub or overestimated.

## 5 Conclusions

The present study showed a correlation between cognitive condition with socio-demographic, and health literacy among elderly people living with DM. Cognitive function is an important factor associated with HL in elderly people with DM, for all elements associated with treatment (physical activity, diet and medication use). In the context of Brazilian public health, the use of instruments/tools to evaluate the HL and cognitive function among people living with DM could improve the effectiveness in the management of the disease. Therefore, interventions aiming to improve the treatment of DM for those with poor HL should also consider the improvement of cognitive condition that is commonly affected by age.

## Informed consent

All volunteers signed an informed consent, according to Brazilian ethical regulations (National Health Council, resolution 466/12) and Declaration of Helsinki. For individuals unable to sign it (for example, individual with dementia), the document was obtained from a guardian. The informed consent was signed in duplicate (copy for researchers and participants).

## Compliance with ethical standards

All experiments were performed according to procedures approved by Ethics Committee of Universidade Estadual de Montes Claros Plataforma Brasil (protocol number 764.743).

## Funding sources

This work was supported by grants from *Coordenadoria de Aperfeiçoamento do Pessoal de Nível Superior* (CAPES), *Conselho Nacional de Desenvolvimento Científico e Tecnológico* (CNPq) and *Fundação de Amparo à Pesquisa de Minas Gerais* (FAPEMIG).

## Conflicts of Interest

The authors declare no conflict of interest.

## References

[bib0005] Al-Hayek A.A., Robert A.A., Alzaid A.A., Nusair H.M., Zbaidi N.S., Al-Eithan M.H., Sam A.E. (2012). Association between diabetes self-care, medication adherence, anxiety, depression, and glycemic control in type 2 diabetes. Saudi Med. J..

[bib0010] Almeida O.P. (1998). Mini mental state examination and the diagnosis of dementia in Brazil. Arq. Neuropsiquiatr..

[bib0015] American Diabetes Association (2010). Diagnosis and classification of diabetes mellitus. Diabetes Care.

[bib0020] American Diabetes Association (2018). 2. Classification and diagnosis of diabetes: standards of medical care in diabetes-2018. Diabetes Care.

[bib0025] Apolinario D., Braga Rde C., Magaldi R.M., Busse A.L., Campora F., Brucki S., Lee S.Y. (2012). Short assessment of health literacy for portuguese-speaking adults. Rev. Saude Publica.

[bib0030] Bailey S.C., Brega A.G., Crutchfield T.M., Elasy T., Herr H., Kaphingst K., Karter A.J., Moreland-Russell S., Osborn C.Y., Pignone M., Rothman R., Schillinger D. (2014). Update on health literacy and diabetes. Diabetes Educ..

[bib0035] Bains S.S., Egede L.E. (2011). Associations between health literacy, diabetes knowledge, self-care behaviors, and glycemic control in a low income population with type 2 diabetes. Diabetes Technol. Ther..

[bib0040] Batista-Jorge G.C., Barcala-Jorge A.S., Silveira M.F., Lelis D.F., Andrade J.M.O., de Paula A.M.B., Guimarães A.L.S., Santos S.H.S. (2020). Oral resveratrol supplementation improves Metabolic Syndrome features in obese patients submitted to a lifestyle-changing program. Life Sci..

[bib0045] Bruce D.G., Casey G.P., Grange V., Clarnette R.C., Almeida O.P., Foster J.K., Ives F.J., Davis T.M., Fremantle Cognition in Diabetes S. (2003). Cognitive impairment, physical disability and depressive symptoms in older diabetic patients: the Fremantle Cognition in Diabetes Study. Diabetes Res. Clin. Pract..

[bib0050] Brucki S.M., Nitrini R., Caramelli P., Bertolucci P.H., Okamoto I.H. (2003). Suggestions for utilization of the mini-mental state examination in Brazil. Arq. Neuropsiquiatr..

[bib0055] Cardoso M., Santos A.S.F., Fonseca A.D.G., Silva-Junior R.F.D., Carvalho P.D., Martins A. (2019). Validity and reliability of the Health Literacy Assessment Scale for adherence to drug treatment among diabetics. Einstein (Sao Paulo).

[bib0060] Chatterjee S., Davies M.J., Heller S., Speight J., Snoek F.J., Khunti K. (2018). Diabetes structured self-management education programmes: a narrative review and current innovations. Lancet Diab. Endocrinol..

[bib0065] Cohen J. (1988). Statistical Power Analysis for the Behavioral Sciences.

[bib0070] Eleutério T.P., Pereira É.J., Farias P.K.S., Hott K.P.S., Paula F.M.Td., Martins A.M.E.B.L. (2018). Elaboração e verificação da validade e confiabilidade de um instrumento de letramento em nutrição entre pessoas com diabetes. Cadernos Saúde Coletiva.

[bib0075] Estrada C.A., Martin-Hryniewicz M., Peek B.T., Collins C., Byrd J.C. (2004). Literacy and numeracy skills and anticoagulation control. Am. J. Med. Sci..

[bib0080] Federman A.D., Sano M., Wolf M.S., Siu A.L., Halm E.A. (2009). Health literacy and cognitive performance in older adults. J. Am. Geriatr. Soc..

[bib0085] Ferri C.P., Prince M., Brayne C., Brodaty H., Fratiglioni L., Ganguli M., Hall K., Hasegawa K., Hendrie H., Huang Y., Jorm A., Mathers C., Menezes P.R., Rimmer E., Scazufca M., Alzheimer’s Disease I. (2005). Global prevalence of dementia: a Delphi consensus study. Lancet.

[bib0090] Folstein M.F., Folstein S.E., McHugh P.R. (1975). “Mini-mental state”. A practical method for grading the cognitive state of patients for the clinician. J. Psychiatr. Res..

[bib0095] Friis K., Vind B.D., Simmons R.K., Maindal H.T. (2016). The Relationship between health literacy and health behaviour in people with diabetes: a danish population-based study. J. Diabetes Res..

[bib0100] Gazmararian J.A., Baker D.W., Williams M.V., Parker R.M., Scott T.L., Green D.C., Fehrenbach S.N., Ren J., Koplan J.P. (1999). Health literacy among Medicare enrollees in a managed care organization. JAMA.

[bib0105] Hamasaki H. (2016). Daily physical activity and type 2 diabetes: a review. World J. Diabetes.

[bib0110] International Diabetes Federation (2019). Diabetes Atlas.

[bib0115] Iwata I., Munshi M.N. (2009). Cognitive and psychosocial aspects of caring for elderly patients with diabetes. Curr. Diab. Rep..

[bib0120] Jorge A.S., Jorge G.C., Paraiso A.F., Franco R.M., Vieira L.J., Hilzenderger A.M., Guimaraes A.L., Andrade J.M., De-Paula A.M., Santos S.H. (2017). Brown and white adipose tissue expression of IL6, UCP1 and SIRT1 are associated with alterations in clinical, metabolic and anthropometric parameters in obese humans. Exp. Clin. Endocrinol. Diabetes.

[bib0125] Joseph J.J., Echouffo-Tcheugui J.B., Golden S.H., Chen H., Jenny N.S., Carnethon M.R., Jacobs D., Burke G.L., Vaidya D., Ouyang P., Bertoni A.G. (2016). Physical activity, sedentary behaviors and the incidence of type 2 diabetes mellitus: the Multi-Ethnic Study of Atherosclerosis (MESA). BMJ Open Diabetes Res. Care.

[bib0130] Lakens D. (2013). Calculating and reporting effect sizes to facilitate cumulative science: a practical primer for t-tests and ANOVAs. Front. Psychol..

[bib0135] Lam M.H., Leung A.Y. (2016). The effectiveness of health literacy oriented programs on physical activity behaviour in middle aged and older adults with type 2 diabetes: a systematic review. Health Psychol. Res..

[bib0140] Lee Y.J., Shin S.J., Wang R.H., Lin K.D., Lee Y.L., Wang Y.H. (2016). Pathways of empowerment perceptions, health literacy, self-efficacy, and self-care behaviors to glycemic control in patients with type 2 diabetes mellitus. Patient Educ. Couns..

[bib0145] Liu X.N., Xia Q.H., Fang H., Li R., Chen Y.Y., Yan Y.J., Zhou P., Yao B.D., Jiang Y., Rothman W.G., Xu W. (2018). Effect of health literacy and exercise-focused interventions on glycemic control in patients with type 2 diabetes in China. Zhonghua Liu Xing Bing Xue Za Zhi.

[bib0150] Mambiya M., Shang M., Wang Y., Li Q., Liu S., Yang L., Zhang Q., Zhang K., Liu M., Nie F., Zeng F., Liu W. (2019). The Play of Genes and Non-genetic Factors on Type 2 Diabetes. Front. Public Health.

[bib0155] Martins A., Bauman C., Ávila W., Farias P., Pereira É., Ferreira F., Santos A., Martins M., Oliveira I., Alcântara V., Silva L., Campos L., Silva M., Cardoso M. (2018). Elaboração de um instrumento de alfabetização em saúde quanto à prática de atividade física entre diabéticos. Revista Eletrônica Acervo Saúde. Esp..

[bib0160] Mendes R., Martins S., Fernandes L. (2019). Adherence to medication, physical activity and diet in older adults with diabetes: its association with cognition, anxiety and depression. J. Clin. Med. Res..

[bib0165] Moura N.D.S., Lopes B.B., Teixeira J.J.D., Oria M.O.B., Vieira N.F.C., Guedes M.V.C. (2019). Literacy in health and self-care in people with type 2 diabetes mellitus. Rev. Bras. Enferm..

[bib0170] Nath C.R., Sylvester S.T., Yasek V., Gunel E. (2001). Development and validation of a literacy assessment tool for persons with diabetes. Diabetes Educ..

[bib0175] Neto E.Z., O. C, Oliveira I.A., Pereira V.B.V., Nogueira J.F.M., Procópio J.P.M., Alcântara V.R.A., Cardoso M.L.F., Silva L.T.S., Farias P.K.S., Martins A.M.E.B.L. (2018). Cross-cultural adaptation and evaluation of psychometric properties of the Literacy Assessment for Diabetes - LAD-60. REAS, Revista Eletrônica Acervo Saúde.

[bib0180] Nguyen H.T., Kirk J.K., Arcury T.A., Ip E.H., Grzywacz J.G., Saldana S.J., Bell R.A., Quandt S.A. (2013). Cognitive function is a risk for health literacy in older adults with diabetes. Diabetes Res. Clin. Pract..

[bib0185] Paim J., Travassos C., Almeida C., Bahia L., Macinko J. (2011). The Brazilian health system: history, advances, and challenges. Lancet.

[bib0190] Rothman R.L., DeWalt D.A., Malone R., Bryant B., Shintani A., Crigler B., Weinberger M., Pignone M. (2004). Influence of patient literacy on the effectiveness of a primary care-based diabetes disease management program. JAMA.

[bib0195] Roundtable on Health Literacy, Board on Population Health and Public Health Practice, Institute of Medicine (2013). Health Literacy: Improving Health, Health Systems, and Health Policy Around the World: Workshop Summary.

[bib0200] Sanders L.M.J., Hortobagyi T., la Bastide-van Gemert S., van der Zee E.A., van Heuvelen M.J.G. (2019). Dose-response relationship between exercise and cognitive function in older adults with and without cognitive impairment: a systematic review and meta-analysis. PLoS One.

[bib0205] Scazufca M., Almeida O.P., Vallada H.P., Tasse W.A., Menezes P.R. (2009). Limitations of the mini-mental state examination for screening dementia in a community with low socioeconomic status: results from the Sao Paulo Ageing & Health Study. Eur. Arch. Psychiatry Clin. Neurosci..

[bib0210] Sorensen K., Van den Broucke S., Fullam J., Doyle G., Pelikan J., Slonska Z., Brand H., Consortium Health Literacy Project E. (2012). Health literacy and public health: a systematic review and integration of definitions and models. BMC Public Health.

[bib0215] Souza A.C., Magalhães Ld.C., Teixeira-Salmela L.F. (2006). Adaptação transcultural e análise das propriedades psicométricas da versão brasileira do Perfil de Atividade Humana. Cadernos de Saúde Pública.

[bib0220] Stumvoll M., Goldstein B.J., van Haeften T.W. (2005). Type 2 diabetes: principles of pathogenesis and therapy. Lancet.

[bib0225] Triola M. (1999). Introdução à Estatística. Rio De Janeiro.

[bib0230] Ueno H., Ishikawa H., Suzuki R., Izumida Y., Ohashi Y., Yamauchi T., Kadowaki T., Kiuchi T. (2019). The association between health literacy levels and patient-reported outcomes in Japanese type 2 diabetic patients. SAGE Open Med..

[bib0235] Yeh J.Z., Wei C.J., Weng S.F., Tsai C.Y., Shih J.H., Shih C.L., Chiu C.H. (2018). Disease-specific health literacy, disease knowledge, and adherence behavior among patients with type 2 diabetes in Taiwan. BMC Public Health.

